# Commentary: “Long-Term Outcomes and Prognostic Analysis of Computed Tomography-Guided Radioactive ^125^ I Seed Implantation for Locally Recurrent Rectal Cancer After External Beam Radiotherapy or Surgery”: Letter to the Editor

**DOI:** 10.3389/fonc.2021.750922

**Published:** 2021-11-30

**Authors:** Enli Chen, Fenxian Zhang, Chenfei Jia

**Affiliations:** ^1^Department of Oncology, Graduate School of Hebei Medical University, Hebei Medical University, Shijiazhuang, China; ^2^Department of Life Science, College of Life Science, Shanxi Agricultural University, Jinzhong, China; ^3^Department of Oncology, Cangzhou Central Hospital, Cangzhou, China

**Keywords:** locally recurrent rectal cancer (LRRC), ^125^ I seed implantation, dosimetry, prognosis, adverse effects

Because ^125^ I seeds can deliver a high dose of radiation to the tumor target while sparing surrounding normal tissues, it is increasingly used for the treatment of various cancer types and has already achieved promising efficacy. In a recent retrospective study, Wang et al. (1) investigated the long-term efficacy and normal tissue toxicities experienced by 101 patients with locally recurrent rectal cancer (LRRC) after external beam radiotherapy or surgery treated by interstitial implantation of ^125^ I seeds. The study is of great significance in the treatment of LRRC, which offers a highly effective treatment. However, there are still some issues that we expect to discuss with the authors.

First, when they applied the Chi-square test and Fisher’s exact test to analyze factors correlated with adverse effects, they did not include the dose of the organ at risk (OAR) such as D2cc or D0.1cc of skin or mucosa. It is well known that the dose of OAR is closely related to the occurrence of radiation damage (2–4). Therefore, we recommend that the authors further analyze the detailed relationship between the dose of OAR and radiation damage. We also hope that the authors can explore the cutoff value of the dose of OAR (when the dose of OAR is greater than this calculated cutoff value, radiation damage is more likely to occur). Such results may be more favorable to the formulation of a preoperative implantation plan to control post-implantation radiation damage. In addition, it is also necessary to include D100, V150, and V200, because D100 can reflect the dose in the peripheral area of the tumor, and V150 and V200 can reflect the volume of the high dose within the tumor target (5, 6). In our recent study (6), we found that the faster the tumor shrank, the more likely the ^125^ I seeds were to gather centrally, which might lead to the local ultra-high dose region, thereby causing radiation damage to surrounding normal tissues. If patients achieve complete remission over short periods, we believe that it is more likely to lead to radiation damage (6, 7). Thus, the short-term efficacy should also be included in the analysis of adverse effects.

Additionally, when they analyzed factors associated with local control (LC), they did not include all observed postoperative parameters such as D100, V150, homogeneity index (HI), conformal index (CI), and external index (EI), which we suppose are of paramount importance. We suggest that the authors include these missing parameters recorded. Another issue is how is the cutoff value calculated? For example, why is D90 grouped by 129 Gy? Why is GTV grouped by 50 cm^3^? Unfortunately, we did not find the grouping basis in the *Method* section of the article. We hold that the most scientific approach is to use X-tile software to determine the cutoff value for all the continuous data (8). Furthermore, as the authors stated, treatment modalities used for LRRC patients after ^125^ I seed implantation were not consistent as well; some patients received postoperative chemotherapy, which, however, was poorly tolerated by other patients. It is well known that the combination of radiotherapy and chemotherapy can contribute to significant improvements in local tumor control and cure rates. Thus, it is recommended to include postoperative chemotherapy for covariate adjustment when the Cox proportional hazards regression model is used for multivariate analysis.

In conclusion, the authors left out some important variables in their analysis of adverse effects, and they failed to include postoperative chemotherapy for covariate adjustment in multivariate analysis of LC. In addition, they did not state the grouping basis of continuous data in the *Method* section of the article. We believe that the most scientific approach is to use X-tile software to determine the cutoff value for all the continuous data.

## Author Contributions

EC, FZ, and CJ wrote the letter. All authors contributed to the article and approved the submitted version.

## Conflict of Interest

The authors declare that the research was conducted in the absence of any commercial or financial relationships that could be construed as a potential conflict of interest.

## Publisher’s Note

All claims expressed in this article are solely those of the authors and do not necessarily represent those of their affiliated organizations, or those of the publisher, the editors and the reviewers. Any product that may be evaluated in this article, or claim that may be made by its manufacturer, is not guaranteed or endorsed by the publisher.
